# Beyond the binary: towards obesity prevention and treatment as a continuum

**DOI:** 10.1093/eurpub/ckag112

**Published:** 2026-07-02

**Authors:** Joreintje D Mackenbach, Harry Rutter

**Affiliations:** Epidemiology and Data Science, Amsterdam UMC Location Vrije Universiteit, Amsterdam, The Netherlands; Amsterdam Public Health, Amsterdam, The Netherlands; Upstream Team, Amsterdam, The Netherlands, www.upstreamteam.nl; Centre for 21st Century Public Health, University of Bath, Bath BA2 7AY, United Kingdom; Department of Nutrition and Public Health, Faculty of Health and Sport Sciences, University of Agder, Kristiansand, Norway

## Introduction

Since their development over the last decade, there has been a rapid uptake of glucagon-like peptide-1 receptor agonists (GLP-1 RAs, or GLP-1 drugs) for the treatment of obesity. The magnitude of their clinical impact has led to concerns that less emphasis may now be placed on obesity prevention, resulting in a reductionist framing in which treatment and prevention are positioned as competing rather than complementary responses to obesity.

In this position paper, we argue that this kind of dichotomous framing is harmful for both patients and population health. It is essential prevention and treatment of obesity are appreciated as complementary components of a shared continuum. Both are essential to manage one of the defining health challenges of our century.

## Treatment and prevention are complementary

Treatment and prevention each address mechanisms that the other cannot. GLP-1 drugs represent a significant breakthrough for the millions of individuals living with obesity today. Clinical trial data show mean body weight reductions of 12%–22% with injectable semaglutide and tirzepatide, respectively [1]. These are outcomes that were previously achievable only through bariatric surgery, and provide greater and more rapid benefits for individual patients than population-level prevention policies.

Yet, the evidence also reveals the limits of pharmacotherapy as a population-level solution. Large real-world cohorts demonstrate that up to half of GLP-1 users discontinue treatment within a year [2]. Weight regain following discontinuation is well-documented [3], and may result in worse cardiometabolic health than before GLP-1 treatment. Gastrointestinal side effects, cost, and comorbidity burden are the primary drivers, suggesting that the proportion of the population who can both tolerate and sustain these medications long-term is considerably smaller than trial efficacy data might suggest [1].

Healthier built environments, including accessible green space, active travel infrastructure, and food retail policies that improve the nutritional quality of available food, all have the potential to help people on pharmacotherapy, or following bariatric surgery, to initiate and sustain healthy behaviour change, and thus maximize treatment response. A patient taking a GLP-1 agonist who lives in a food environment dominated by cheap, heavily marketed unhealthy foods faces greater challenges than one living in a community characterized by easy access to fresh, affordable food. Even if GLP-1 agonists were better tolerated and more effective, populations would continue to benefit considerably from environments, policies, and behaviours that support healthy behaviours and healthy weight.

Effective treatments and prevention have always coexisted. Antihypertensive medications are among the most prescribed drugs in the world, yet clinical guidelines advise patients also to limit sodium and alcohol intake and increase physical activity. Similarly, statins substantially reduce cardiovascular risk, yet cardiac prevention guidelines continue to emphasize diet, physical activity, and smoking cessation alongside pharmacotherapy. Antiretroviral therapy has made HIV a chronic manageable condition for many individuals, yet public health investment in prevention, such as condom promotion, needle exchange, and pre-exposure prophylaxis programmes, has not been abandoned. In each of these cases, treatment and prevention operate in parallel, each addressing mechanisms that the other cannot, acting synergistically to improve health.

Obesity is no different. The biological mechanisms that GLP-1 drugs act upon, dysregulated appetite signalling and impaired satiety, are also affected by food, physical activity, economic and social environments—the structural conditions in which people live [4]. Medication does not change the obesogenic environment; prevention efforts do. At the same time, prevention efforts alone may be inadequate to reverse the individual-level physiological changes already present in a person with established obesity.

A lifecourse perspective on obesity reinforces that future generations would also benefit considerably from healthy environments. Obesity tracks strongly from childhood into adulthood, so preventing it from arising in the first place, or reducing its severity if it does develop, may carry lifelong benefits. Robust action to reduce the incidence of childhood obesity thus has the potential not only to have major benefits over the lifecourse, it can also reduce the costs and risks associated with lifelong pharmacotherapy.

## The need for multiple, multilevel responses

Addressing obesity effectively requires action at multiple levels at the same time. It is not enough to direct treatment and prevention efforts only at individuals, whether through pharmacotherapy, behavioural support, or clinical guidance. The food environments, built environments, and commercial systems in which people live shape behavioural risk factors in ways that individual-level interventions cannot fully address. Population-level policies, such as food taxes, marketing restrictions and building active travel infrastructure, are an integral part of prevention, not an afterthought to it [5].

No single intervention, however effective in isolation, is sufficient on its own to address obesity [6]. Smallpox eradication was driven by vaccination, but supported by isolation and other measures to reduce exposure. Smoking cessation programmes did not end lung cancer; antihypertensive drugs did not end strokes; and seat belt legislation did not end road fatalities. In each case, meaningful progress came from a range of measures deployed in parallel and at multiple levels—and the same logic applies to obesity.

A further argument for combining multiple preventive and curative strategies comes from systems science. Obesity is widely recognized as a complex systems problem, driven by nonlinear interactions between biological, behavioural, commercial, and other structural forces [7]. Interventions in complex systems reliably produce unintended consequences: not as exceptional outcomes, but as an inherent feature of dynamic systems [8]. The introduction of mass pharmacotherapy into the obesity system is no exception. Every intervention, however effective, carries risks of harms that can ripple through the wider system in ways that are difficult fully to anticipate, but which may nevertheless be amenable to mitigation.

Several examples illustrate this. Taxes on unhealthy foods can reduce consumption but risk being financially regressive; this can be mitigated by pairing them with healthy food subsidies or cash transfer mechanisms that offset the burden on lower-income households [9]. Front-of-pack warning labels can drive reformulation but may be circumvented by industry relabelling or product repositioning, underscoring the need for complementary regulatory measures. Mass pharmacotherapy raises serious environmental concerns: producing GLP-1 drugs requires toxic organic solvents, and plastic injection pens contribute to water and soil pollution. Injection device disposal adds a further waste burden that the current healthcare infrastructure is poorly equipped to manage. These concerns will only grow as GLP-1 medications come down in price and become affordable to an increasing proportion of the more than one billion people living with obesity worldwide.

There may even be further downstream consequences of mass pharmacological weight reduction. Life insurance underwriting models, pension liability calculations, and infrastructure design have all been calibrated to a world with high obesity prevalence, and may shift in response to large-scale population weight loss in ways that are currently difficult to model. The profits of the food and bariatric surgery industries may be affected also: GLP-1 uptake has been proposed as a cause of declining sugar prices and a measurable reduction in bariatric procedures.

No single intervention is protected from such consequences. The value of combining curative and preventive efforts at multiple levels is that doing so distributes risk across multiple strategies.

Finally, it is important to remain alert to whose interests are served by a predominantly clinical framing of obesity. The ways in which obesity is framed, and the responses proposed to address it, have been strongly influenced by media and other commercial actors. The growing movement to classify obesity as a chronic disease has clinical merit and may have helped reduce the stigma associated with personal blame. However, research has documented how leading GLP-1 manufacturers have strategically constructed obesity as a biomedical problem requiring pharmaceutical intervention, while simultaneously positioning themselves as advocates for patients and opponents of stigma [10]. The aggressive promotion of GLP-1 drugs on social media reflects a broader process of pharmaceuticalization, in which complex social and structural problems are increasingly reframed as medical conditions amenable to pharmaceutical solutions. It is therefore important to ensure that the case for prevention, offering far less commercial reward, is made with continued vigour. Only about 3% of overall health spending across OECD countries is currently spent on prevention. It is thus especially important to ensure that pharmacological innovation does not further marginalize the already underfunded case for prevention.

## Conclusion

GLP-1 drugs offer a valuable therapeutic option for patients with obesity today. Prevention offers the prospect of fewer people developing obesity tomorrow while also helping those already living with obesity. It is essential that policymakers, clinicians, public health professionals, and others understand prevention and treatment of obesity as complementary components of a shared continuum. Increased attention for pharmacotherapy should not come at the expense of policy attention and public investment in structural prevention efforts. The need for effective obesity prevention policies remains, alongside that for expanded access to treatment, and all solutions should be monitored for unintended consequences.

Key points

- Treatment and prevention should be viewed as complementary responses to obesity.
- This is because they operate synergistically, reaching different populations and risk factors, and complementary, enhancing each other and mitigating each other’s unwanted effects.
- Yet, commercially driven attention for pharmacological innovations should not further marginalize prevention efforts, which already receive inadequate levels of both funding and policy attention.

## Data Availability

Not applicable.
